# Comparative Metabolomic and Transcriptomic Analyses Identify Candidate Genes Associated with Flavonoid Accumulation and Phenylpropanoid Metabolism in Large-Fruited Hawthorn (*Malus doumeri* (Bois) Chev.)

**DOI:** 10.3390/molecules31111857

**Published:** 2026-05-28

**Authors:** Xiao-Hua Dai, Xiang-Ying Wei, Lu-Xia Ran, Jing Chen, Feng-Jin Zheng, Usman Rasheed, Gan-Lin Chen

**Affiliations:** 1 Guangxi Subtropical Crops Research Institute, Guangxi Academy of Agricultural Sciences, Nanning 530001, China; xiaohuadai2023@163.com (X.-H.D.); 15102932953@163.com (X.-Y.W.); 1211016008@gnnu.edu.cn (L.-X.R.); jchen1030@gxaas.net (J.C.); rasheus@outlook.com (U.R.); 2 Key Laboratory of Quality and Safety Control for Subtropical Fruit and Vegetable, Ministry of Agriculture and Rural Affairs, Nanning 530001, China; 3 Guangxi Key Laboratory of Quality and Safety Control for Subtropical Fruits, Nanning 530001, China; 4 Institute of Agro-Products Processing Science and Technology, Guangxi Academy of Agricultural Sciences, Nanning 530007, China

**Keywords:** RNA-seq, metabolomics, metabolite biosynthesis, *Malus doumeri* (Bois) Chev., large-fruited hawthorn

## Abstract

Large-fruited hawthorn (*Malus doumeri* (Bois) Chev.) is valued for its health-promoting properties, largely attributed to its rich flavonoid content. However, little is known about the specific composition of flavonoids and the molecular mechanisms regulating their biosynthesis. The present study employed non-targeted metabolomic and transcriptomic approaches to investigate two *M. doumeri* germplasms (G8 and G9) that exhibited significantly different total flavonoid contents. The results indicated that the major and differential metabolites primarily include flavonoids and isoflavonoids. Differentially expressed genes were significantly enriched in phenylpropanoid and flavone and flavonol biosynthesis pathways. Integrated analysis identified several structural genes and transcription factors, including *HCT* (LOC114821133, LOC103403337, LOC103454980), *WRKY* (LOC103427630), and *bHLH* (LOC103422512), that were significantly upregulated in the high-flavonoid genotype (G9). qRT-PCR validation confirmed the RNA-Seq expression patterns, suggesting the potential involvement of these genes in the biosynthesis of phenylpropanoid-related metabolites, such as [6]-gingerol. Applied experiments further demonstrated that freeze-drying preserved high metabolite contents and antioxidant activity. Collectively, these findings provide insights into the compositional characteristics of the major flavonoids in *M. doumeri* and the biosynthesis of phenylpropanoid-derived metabolites. This study provides data support for future mechanistic validation and evaluation of processing technology applicability.

## 1. Introduction

*Malus doumeri* (Bois) Chev., commonly known as large-fruited hawthorn, is a species of the family Rosaceae and genus *Malus*, predominantly distributed in southern China, Vietnam, and Laos [1]. Jingxi County in Guangxi, China, has the longest history of cultivation, the widest distribution, and the largest cultivation area among these regions. It also produces the biggest fruit size, leading to the species of *M. doumeri* being recognized as a geographical indication product [2]. *M. doumeri* serves dual purposes, offering medicinal and nutritional benefits. Its fruit is consumed fresh, processed into preserved products, or used in winemaking, while its leaves are often used in tea making. The dried plant is used in traditional medicine and has been reported to possess several pharmacological activities. These include regulating vital energy, enhancing spleen and stomach function, promoting digestion, and exhibiting notable antibacterial and antioxidant effects [3,4,5]. It has been officially recognized and included in the standards of the Chinese Pharmacopoeia [6]. With the increasing market value of *M. doumeri*, its use and development have become significant drivers of local industrial growth. Thus, conducting further in-depth research is scientifically valuable and strategically essential for promoting sustainable resource development and advancing regional economy.

Previous studies have mainly examined the phytochemical and pharmacological properties of *M. doumeri* fruits, leaves, and processed products, using extraction-assisted preparation methods followed by HPLC-based analysis. These components include flavonoids, triterpenes, phenolics, amino acids, organic acids, and vitamins. Comparative analyses of different maturation stages and geographic origins have been performed, including assessments of antioxidant activity and antibacterial efficacy. The findings indicate significant variations in functional compounds among different germplasms [3,4,5,7,8,9,10]. However, investigations at the molecular level to unravel the mechanisms behind the biosynthesis of significant compounds are still limited. Currently, integrating metabolomics with transcriptomics to elucidate the molecular mechanisms underlying metabolite formation is an effective and widely adopted research strategy [11]. In cultivated apple (*Malus domestica*), extensive studies have investigated the biosynthetic mechanisms of metabolites associated with flavor, color, stress resistance, and bioactivity [12,13,14,15]. North hawthorn (*Crataegus pinnatifida*), which is often confused with *M. doumeri*, has also been extensively studied for its physicochemical properties, functional metabolites, coloration, and bioactivity [16,17,18,19,20,21,22]. Utilizing multi-omics approaches to elucidate the molecular mechanisms and regulatory pathways involved in the biosynthesis of essential functional constituents would establish a solid theoretical framework for the genetic manipulation of this species.

Plants contain a variety of flavonoid compounds that are essential for pigmentation, flavor, regulating cell growth, attracting pollinators, and enhancing resistance to environmental stresses. When consumed by humans, flavonoids exhibit a variety of health benefits and pharmacological effects, including antioxidant, anti-inflammatory, and immunomodulatory properties. Owing to their unique bio-functional properties, flavonoids are widely used in the food, cosmetics, and pharmaceutical industries [23,24,25]. The biosynthetic pathways and molecular regulatory mechanisms of flavonoids have been extensively characterized in various plant species [26]. In recent years, identification and screening of several key regulatory modules involved in flavonoid biosynthesis in Rosaceae fruits have been performed using transcriptomics, metabolomics, and proteomics technologies and validated through qRT-PCR, Agrobacterium-mediated transient expression, luciferase reporter assay, and transient GUS activity assay. In contrast, research on M. doumeri has largely focused on phytochemical characterization and pharmacological activity assays, whereas the molecular basis of functional metabolite biosynthesis remains poorly understood. This knowledge gap limits the comprehensive assessment of its nutritional value and constrains efforts toward targeted breeding and product development. The application of integrative multi-omics provides a promising approach for identifying candidate genes and pathways associated with metabolite buildup in this species, thereby supporting its informed use.

Given the existing literature and identified research gaps, this study integrated metabolomic and transcriptomic approaches to compare two *M. doumeri* germplasms differing in total flavonoid content and to preliminarily assess the effects of processing on the identified functional metabolites. The findings of this study may provide a reference for the nutritional evaluation and germplasm selection of the large-fruited hawthorn (*M. doumeri*) in Guangxi, China, as well as for the further development of health-promoting products.

## 2. Results

### 2.1. Subsection Total Flavonoid Content and Metabolomic Profiling

**Figure 1:**
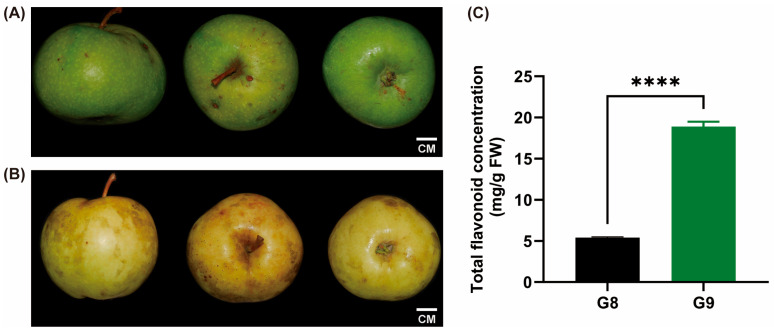
Comparison of the mature fruits of *M. doumeri* germplasms G8 (**A**) and G9 (**B**), and comparison of total flavonoid content between G8 and G9 (**C**). Data are presented as mean ± SEM (*n* = 3 biological replicates per group). Statistical significance **** using Student’s t-test indicates *p* < 0.0001.

**Figure 2:**
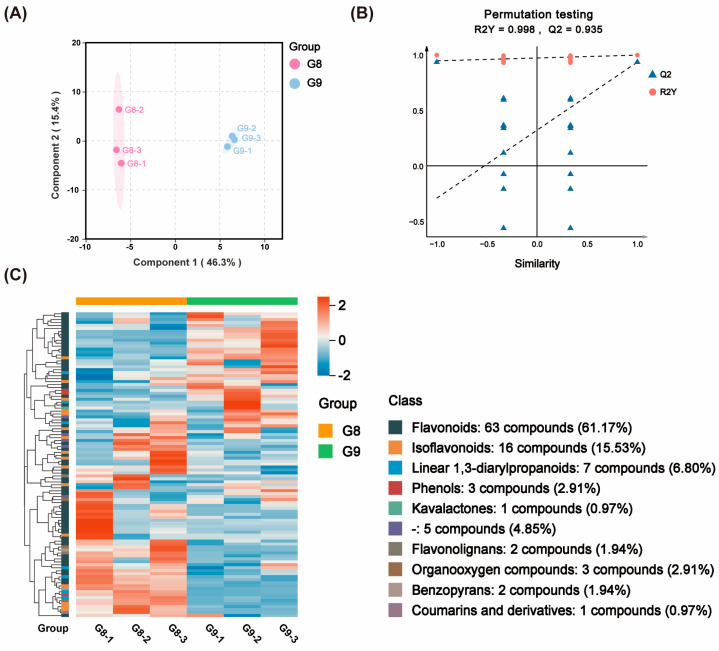
Multivariate statistical analysis of metabolites in G8 and G9. OPLS-DA score plot (**A**), permutation test of the OPLS-DA model (**B**), and hierarchical cluster analysis (HCA) of metabolite abundance across the samples annotated by subclass (**C**). G8-1, G8-2, G8-3 and G9-1, G9-2, G9-3 represent three biological replicates per group.

This study detected a total flavonoid content of 18.88 mg/g in fresh G9 fruit, which is 3.5 times higher than G8 (Figure 1). To further understand the metabolic composition, the metabolomic profiling in G8 and G9 revealed a total of 103 metabolites, which included 63 flavonoids (61.17%), 16 isoflavonoids (15.53%), and 7 linear 1,3-diarylpropanoids (6.80%) (Figure 2C). Orthogonal partial least squares discriminant analysis (OPLS-DA) results demonstrated distinct separation between G8 and G9, with R^2^Y and Q^2^ values exceeding 0.9, indicating strong explanatory and predictive performance for the model (Figure 2A,B). Hierarchical cluster analysis (HCA) revealed significant differences in the relative abundance of metabolites between the two germplasms (Figure 2C).

**Figure 3:**
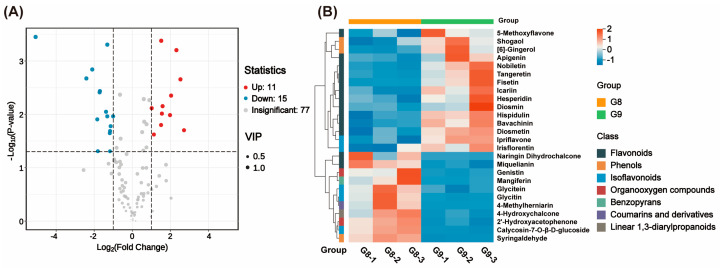
Multivariate statistical analysis of differentially accumulated metabolites (DAMs) between G8 and G9 groups. Volcano plot showing the count of DAMs (**A**), and hierarchical cluster analysis (HCA) of DAMs (**B**). G8-1, G8-2, G8-3 and G9-1, G9-2, G9-3 represent three biological replicates per group.

Based on the criteria of VIP ≥ 1, |log_2_FC| ≥ 1, and *p* < 0.05, identified a total of 26 differentially accumulated metabolites (DAMs) between G8 and G9. This included 11 upregulated and 15 downregulated compounds (Figure 3A). The significant upregulation of metabolites was observed in G9, which included 13 flavonoids, 5 isoflavonoids, 3 phenols, 2 organooxygen compounds, and 1 compound each from the classes of benzopyrans, coumarins and their derivatives, and linear 1,3-diarylpropanoids (Figure 3B).

### 2.2. Transcriptomic Analysis and Differential Gene Expression Profiling

**Figure 4:**
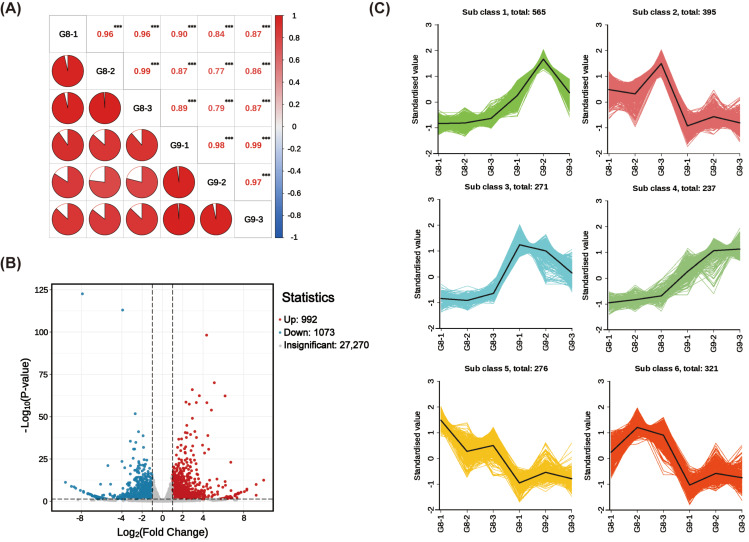
Multivariate statistical analysis of transcriptomic data from G8 and G9 samples. Heatmap of correlation analysis among biological replicates, statistical significance *** indicates *p* < 0.001 (**A**), volcano plot showing significantly differentially expressed genes between G8 and G9 (**B**), and K-means clustering of DEGs, illustrating expression patterns across samples (**C**). G8-1, G8-2, G8-3 and G9-1, G9-2, G9-3 represent three biological replicates per group.

An average of 46,837,115 clean reads was obtained from the G8 and G9 libraries, with an overall clean read rate of 99.77%. The Q30 scores exceeded 95% across all libraries while the average GC content remained 47.02%. On average, 81.49% of the high-quality reads were aligned with the reference genome. This alignment rate indicates substantial sequence conservation and supports the reliability of the transcriptomic analysis (Table S2). To identify differentially expressed genes (DEGs), we performed preliminary screening and correlation analysis on biological replicates. Correlation analysis showed R^2^ values of approximately 0.9 among biological replicates, indicating good reproducibility and supporting their suitability for downstream analysis. (Figure 4A). Using the criteria |log_2_FC| ≥ 1 and adjusted *p*-value < 0.05, a total of 2065 differentially expressed genes (DEGs) between G8 and G9 were identified. Among them, 992 genes were upregulated and 1073 downregulated (Figure 4B). K-means clustering categorized the DEGs into six distinct clusters, showing significant expression differences among biological replicates across all samples (Figure 4C).

**Figure 5:**
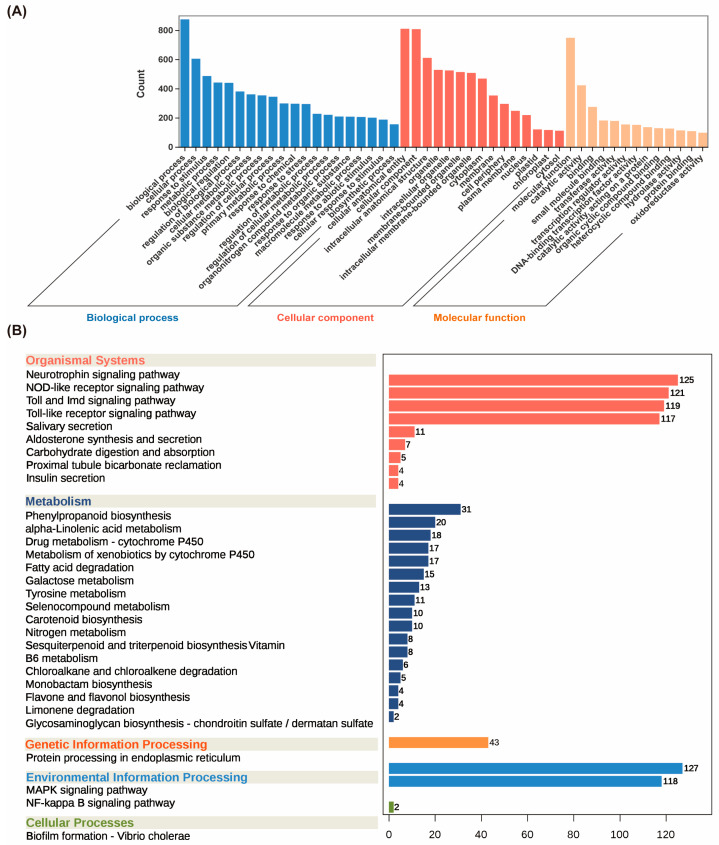
Functional annotation and enrichment analysis of differentially expressed genes between G8 and G9. GO enrichment analysis of DEGs (**A**), and KEGG pathway enrichment analysis of DEGs (**B**).

Differentially expressed genes (DEGs) were subjected to functional annotation and enrichment analysis. Gene Ontology (GO) annotation indicated that the DEGs were mainly enriched in the categories of biological and cellular process within biological process, cellular component within cellular component, and molecular function and catalytic activity within molecular function (Figure 5A). KEGG pathway analysis showed that DEGs were mainly enriched in pathways, such as the MAPK signaling pathway, NF-kappa B signaling pathway, and Neurotrophin signaling pathway. Notably, the DEGs were significantly enriched in phenylpropanoid biosynthesis and flavone and flavonol biosynthesis pathways (Figure 5B).

### 2.3. Differential Expression of Key Regulatory Genes and Associated Pathways

Key differentially expressed genes (DEGs) involved in relevant metabolic pathways were identified based on KEGG pathway enrichment and gene functional annotation (Table S3). In the phenylpropanoid biosynthesis pathway, 28 DEGs were detected, including 2 *4CL*, 3 *CCR*, 2 *CAD*, 4 *COMT*, 4 *K22395*, 5 *E1.11.1.7*, 1 *UGT72E*, 1 *F5H*, and 6 *HCT*. Among these genes, 1 *4CL* gene was significantly upregulated in G9. In contrast, 2 *CCR*, 2 *CAD*, 3 *COMT* genes, 2 *K22395* genes, 1 *E1.11.1.7* gene, and 1 *UGT72E* gene were significantly downregulated in G9. In the flavonoid biosynthesis pathway, 17 DEGs were identified, including 6 *HCT*, 4 *C12RT1*, 3 *FLS*, 1 *LAR*, and 2 *PGT1* genes. 7 genes, including 6 *HCT* genes and 1 *LAR* gene, were significantly upregulated in G9. In the isoflavonoid biosynthesis pathway, 2 DEGs were identified, such as 1 *CYP81E* and 1 *PTS*, with *PTS* showing significant upregulation in G9. In the biosynthesis pathway of flavones and flavonols, 4 DEGs were identified, all annotated as *C12RT1*, and all were significantly downregulated in G9.

### 2.4. Integrated Transcriptomic and Metabolomic Analysis

**Figure 6:**
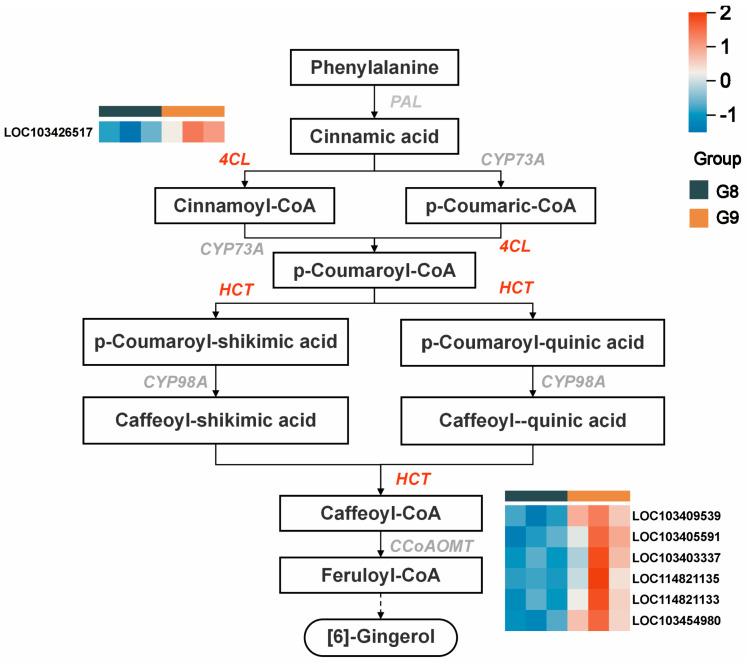
Proposed biosynthetic pathway leading to [6]-gingerol, constructed based on the KEGG reference pathways ko00945 and ko00940, and expression heatmap of associated upregulated genes. Key enzymes: *PAL*, *4CL*, *CYP73A*, *HCT*, *CCoAOMT*, *CYP98A*. Enzymes marked in red indicate significantly upregulated genes in G9. Enzymes marked in grey represent genes with no significant differential expression and heatmap displays relative expression levels of corresponding genes in G8 and G9 (*n* = 3 biological replicates per group).

To gain thorough insights into metabolite biosynthesis, an integrated analysis of DEGs and DAMs was performed. According to the KEGG database, genes and metabolites that appeared to be associated with same metabolic pathways were correlated. The results indicated that the DAMs mainly enriched in five pathways, i.e., flavonoid biosynthesis, isoflavonoid biosynthesis, flavone and flavonol biosynthesis, stilbenoid, diarylheptanoid, and gingerol biosynthesis, and the degradation of flavonoids (Table S4). Interestingly, [6]-gingerol, a differential metabolite with higher abundance in G9, was co-enriched with six *HCT* genes (*LOC103409539*, *LOC103405591*, *LOC103403337*, *LOC114821135*, *LOC114821133*, and *LOC103454980*) in the stilbenoid, diarylheptanoid, and gingerol biosynthesis pathway. These *HCT* genes were also enriched in the flavonoid and phenylpropanoid biosynthesis pathways, were consistently upregulated in G9, and showed strong correlations with [6]-gingerol abundance (*r* > 0.8, *p* < 0.05 for five genes) (Figure 6 and Table S4).

### 2.5. Transcription Factor Profiling

**Figure 7:**
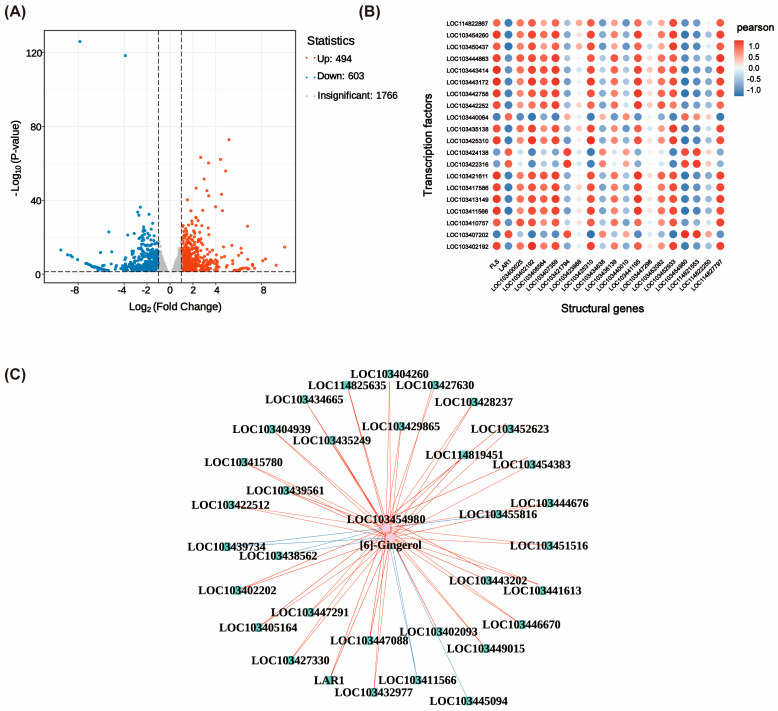
Multivariate statistical analysis of differentially expressed transcription factors between G8 and G9. Volcano plot of differentially expressed transcription factors (**A**), Co-expression correlation analysis between transcription factors and structural genes in associated pathways (**B**), and correlation network of transcription factors (outer ring) significantly associated with selected *HCT* (LOC103454980) and [6]-gingerol (central), retaining only strong (|cor| > 0.9) and statistically significant (*p* < 0.05) associations. Red and blue lines representing positive and negative correlations, respectively (**C**).

A total of 1097 transcription factors (TFs) were identified as differentially expressed between G8 and G9, with 494 being upregulated and 603 downregulated (Figure 7A). Co-expression relationships between transcription factors and structural genes associated with related biosynthetic pathways were explored by correlation analysis of structural genes significantly upregulated in G9. Genes were selected based on the correlation coefficient threshold of *r* > 0.90 and *p* < 0.01 (Figure 7B). Correlation analysis between transcription factors and metabolites identified three transcription factors, i.e., *WRKY* (LOC103427630), *MYB* (LOC103434665), and *bHLH* (LOC103422512). These factors were significantly correlated with *HCT* (LOC103454980) and [6]-gingerol at a significance threshold of *p* < 0.01 [6]-gingerol (Figure 7C and Table S5).

### 2.6. qRT-PCR Validation of the Transcriptomic Data and [6]-Gingerol Content

**Figure 8:**
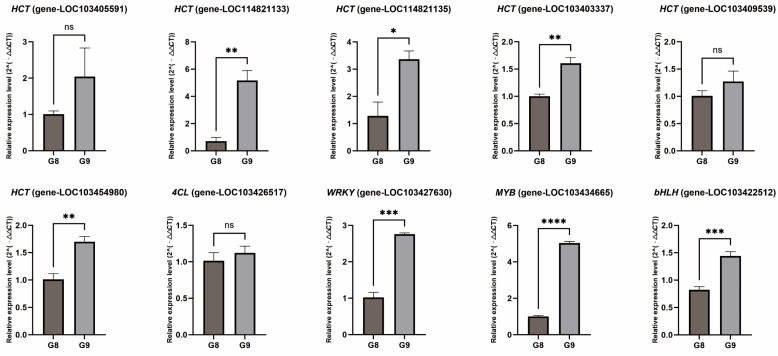
qRT-PCR validation of representative genes of G8 and G9. Data are presented as mean ± SEM (*n* = 3 biological replicates per group), and statistical significance indicates * *p* < 0.05, ** *p* < 0.01, *** *p* < 0.001, **** *p* < 0.0001, ns, not significant.

To validate the key findings from the transcriptome sequencing (RNA-Seq) results, we selected ten DEGs for qRT-PCR analysis. The expression levels of selected genes in G8 and G9 were shown in Figure 8. The expression trends of all tested genes were consistent with the RNA-Seq data, with most showing higher expression in G9. In particular, *HCT* (LOC114821133, LOC103403337, LOC103454980), *WRKY* (LOC103427630), and *bHLH* (LOC103422512) showed the most pronounced upregulation in G9 and a strong correlation between qRT-PCR and RNA-Seq results (*r* > 0.8, *p* < 0.05; Table S6).

### 2.7. Preliminary Evaluation of Processing Effects on Functional Metabolites in M. doumeri

**Figure 9:**
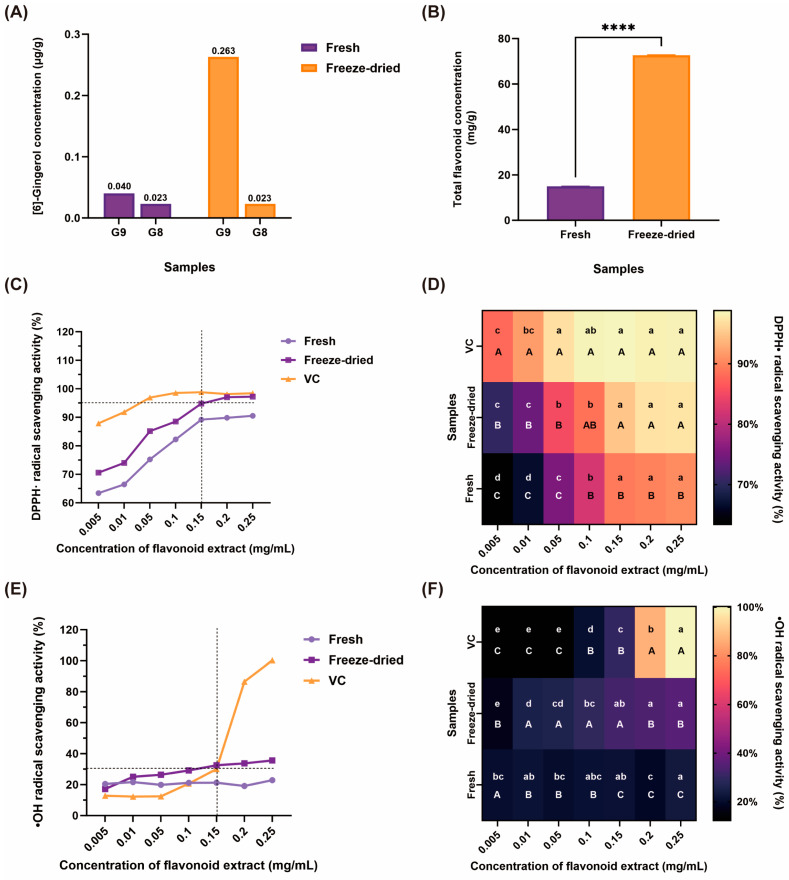
Comparison of [6]-gingerol content between fresh and freeze-dried samples of G8 and G9 (**A**), comparison of total flavonoid content between fresh and freeze-dried of G9 (**B**). Data are presented as mean ± SEM (*n* = 4 biological replicates per group), statistical significance **** using Student’s *t*-test indicates *p* < 0.0001. Comparison of DPPH· (**C**) and ·OH (**E**) radical scavenging activity at different concentrations of total flavonoid extract, and compared the significance of DPPH· (**D**) and ·OH (**F**) radical scavenging activity. Different lowercase letters indicate significant differences in scavenging activity among different total flavonoid concentrations within the same sample in Tukey’s multiple comparison test (*p* < 0.05). Different capital letters indicate significant differences in scavenging activity among samples with the same total flavonoid concentration in Tukey’s multiple comparison test (*p* < 0.05).

Considering the necessity for subsequent application and processing within the food system, we confirmed the absolute quantification of [6]-gingerol using LC-MS. After pretreatment, the [6]-gingerol content in G9 and G8 was measured to be 0.263 and 0.023 μg/g (Freeze-dried), 0.040 and 0.023 μg/g (Fresh), respectively. Based on the analysis, the [6]-gingerol peak area ratio of G9 to G8 was approximately 2, which had a similar trend to the absolute quantification results (Figure 9A and Table S7).

Considering the common practice of sample drying in actual production and processing, we quantified total flavonoid content in G9 fresh and freeze-dried samples, obtaining 14.94 and 72.61 mg/g, respectively (Figure 9B). Furthermore, antioxidant activity was evaluated by assessing DPPH· and ·OH radical scavenging activity, and compared with the potent antioxidant VC (Figure 9C–F). At extract concentrations ≥ 0.10 mg/mL, DPPH· radical scavenging activity exceeded 80%. Particularly for freeze-dried samples, the radical scavenging activity reached 94.77 ± 0.76% at an extract concentration of 0.15 mg/mL, showing no significant difference from VC at the same concentration. In ·OH radical scavenging experiments, when the extract concentration was below 0.15 mg/mL, the scavenging activity of freeze-dried samples exceeded 30%, significantly higher than that of VC. Both DPPH· and ·OH radical scavenging activity increased with rising concentrations of the total flavonoid extract. However, when the concentration was increased to 0.15–0.2 mg/mL, no further increase in the scavenging activity was observed.

## 3. Discussion

### 3.1. Characterization and Implications of Flavonoids in M. doumeri

*M. doumeri* is valued as both a medicinal and edible plant, largely because it is rich in diverse functional metabolites. Its total phenolic content can reach 70–100 mg/g on a dry-weight basis, which is substantially higher than that reported for common fruits such as cultivated apple, hawthorn, and strawberry [3]. Flavonoids represent the predominant class of phenolic compounds in most *M. doumeri* germplasm, with mature fruits containing approximately 15–40 mg/g on a fresh-weight basis [8,27]. This study detected a total flavonoid content of 18.88 mg/g in fresh G9 fruit, which is 3.5 times higher than G8, showing variation among germplasm. To systematically characterize the flavonoid composition of *M. doumeri*, we employed UPLC-MS to identify multiple subclasses including flavonoids, isoflavonoids, and linear 1,3-diarylpropanoids. These results align with previous findings in the leaves of *M. doumeri*, where the primary flavonoid metabolites were identified as flavonoids and linear 1,3-diarylpropanoids. These two classes represented more than 85% of the total peak area in HPLC chromatograms [4]. Similarly, the fruit pulp and leaves of the Rosaceae Malus plants exhibited comparable metabolic profiles [12,28]. This study provides valuable insights into the flavonoid metabolite composition of *M. doumeri* fruits, highlighting a significant relationship between the flavonoid profiles found in the fruits and leaves.

Further analysis indicated that the majority of the upregulated compounds in G9 were identified as flavonoids. This suggests that G9 may be more conducive to the accumulation of specific flavonoid compounds. Flavonoids are essential dietary nutrients that the human body cannot produce on its own. The recommended daily intake of flavonoids ranges from 50 to 800 mg, which can be obtained mainly from fruits, vegetables, tea, and wine [29]. Natural antioxidants have been shown to effectively prevent cardiovascular and neurodegenerative diseases. In comparison to synthetic antioxidants, natural flavonoids are generally safer [23,24]. In Rosaceae fruits like cultivated apple, hawthorn, plum, and peach, flavonoids have also been identified as one of the main secondary metabolites. Their composition and abundance vary depending on cultivar and processing method, thereby contributing substantially to fruit nutritional quality [17,20,21,22,30].

Our research deepens the understanding of flavonoid metabolites in *M. doumeri* fruits. Notably, certain germplasms, like G9, may offer greater nutritional value due to higher levels of specific flavonoids, particularly those that are upregulated. These findings lay the groundwork for further study of the bioactive components in *M. doumeri* fruits, supporting the development of natural flavonoid-rich products, functional foods, and pharmaceuticals. It should be noted that this study selected two germplasms with highly contrasting flavonoid levels, determined using the aluminum nitrate colorimetric method, a widely used approach for preliminary phenotypic screening, to amplify metabolic and transcriptional differences between them. To evaluate the generalizability of these findings, further validation across a broader germplasm collection using complementary, more selective methods is needed.

### 3.2. Transcriptional and Metabolic Insights from Integrated Analysis

Functional annotation and enrichment analysis indicated that the DEGs were mainly enriched in pathways like phenylpropanoid biosynthesis and flavone and flavonol biosynthesis. Phenylpropanoid biosynthesis is a key pathway in plant secondary metabolism that provides essential precursors and structural components for flavonoid biosynthesis [31]. Initiated from phenylalanine, this pathway produces p-coumaroyl-CoA through the sequential involvement of *PAL*, *C4H*, and *4CL*. This intermediate subsequently enters various downstream pathways, including flavonoid, flavone, flavonol, stilbenoid, diarylheptanoid, and gingerol biosynthesis [32], or contributes to the synthesis of lignins and coumarins. The biosynthesis of flavonoids requires structural genes and transcription factors for proper regulation of enzyme expression [32]. Key transcription factor families involved include *MYB*, *bHLH*, *WRKY*, and *AP2*. Among these, the *MYB* family is crucial for regulating flavonoid biosynthesis. *MYB* proteins frequently form complexes with *bHLH* and *WD40* proteins, working together to activate structural genes, such as *FLS* and *F3H*, which enhance the accumulation of flavonols and related compounds [33].

Elevated accumulation of flavonoid compounds can be achieved by enhancing the expression of key enzymes [34], suppressing competing pathways [35], or regulating transcription factors [32]. Previous studies have shown that the Md*MYB*16/Md*MYB*1-miR7125-Md*CCR* module coordinately regulates the light-induced dynamic balance between anthocyanin and lignin biosynthesis in apple [36]. The R2R3-*MYB* transcription factor Gm*MYB*12B2 affected the expression levels of key flavonoid biosynthetic genes *CHI*, *F3H*, and *F3′H* in transgenic *Arabidopsis* plants [37]. In response to *Alternaria alternata* stress, *Korla fragrant* pear enhances flavonoid production by boosting the activity and gene expression of enzymes like *CHS* and *CHI*, leading to increased levels of naringenin, apigenin, and rutin during mid-storage [38]. Under stress conditions, *WRKY* transcription factors can increase the expression of genes, such as *HCT* and *CHS*, which enhances the production of antimicrobial flavonoids. At the same time, *WRKY*s may also suppress specific secondary metabolic branches to optimize resource allocation for defense system [39]. The *SCL8* acts as a negative regulator of *FLS1*, influencing flavanol accumulation in apples [40]. The miR172 indirectly controls *MYB*10-mediated flavonoid synthesis by suppressing *AP2* transcription factor expression [41].

### 3.3. Validation of Transcriptomic and Metabolomic Findings

Based on the above analyses, we speculate that several structural genes and transcription factors identified in this study may be involved in the biosynthesis of [6]-gingerol and related metabolites. To verify this, ten candidate genes were selected for qRT-PCR validation, and the results confirmed the expression patterns observed in the RNA-seq data. In parallel, absolute quantification of [6]-gingerol revealed significantly higher levels in G9 than in G8, consistent with the metabolomic profiling results. Gingerol is a key bioactive metabolite in ginger, known for its medicinal properties, including anti-inflammatory, antioxidant, antitumor, antiemetic, and antinausea effects. It also acts as a natural flavoring agent in the food industry. Its bioactivity, mechanisms of action, and toxicity have been thoroughly evaluated in in vitro and in vivo models, including studies on animals and humans [42,43,44,45]. Although the presence and functional role of [6]-gingerol in fruits remain largely unexplored. Recent findings have reported its occurrence in a few Rutaceae species. Afifi et al. [46] used high-resolution electrospray ionization quadrupole time-of-flight mass spectrometry (nLC-ESI-qTOF-MS) to identify gingerol for the first time in the peel of Citrus sinensis, where it was also found to be abundant in the juice. F. Wang et al. [47] identified gingerol variants in the peel of “Shatangju” Citrus reticulata using UHPLC-QQQ-MS, with levels varying across color phenotypes influenced by Huanglongbing infection. Zollapi et al. [48] identified [6]-gingerol as one of the active ingredients in miracle berry (*Synsepalum dulcificum*) leaf, which can effectively inhibit the target enzyme of diabetes. This finding indicates that [6]-gingerol, previously reported mainly in *Zingiber* species, is also detectable in *M. doumeri* fruit, with abundance varying between germplasms. Its presence in *M. doumeri* may provide a reference for future exploration of this compound beyond its known botanical sources.

*HCT* is a crucial enzyme in the phenylpropanoid biosynthetic pathway which catalyzes the acyl transfer reaction between hydroxycinnamoyl-CoA and shikimic or quinic acid to produce ester intermediates [31]. These intermediates subsequently participate in the biosynthesis of anthocyanins, lignin, phenolic acids, and other related compounds [49,50,51,52,53]. Upregulating *HCT* increases anthocyanin content in blackberry while reducing the synthesis of other secondary metabolites, like lignans [49]. Blue light can induce *HCT* expression, thereby promoting chlorogenic acid biosynthesis in strawberry [52]. In rice, expression downregulation achieved using a truncated *HCT* construct lacking a transcription termination sequence led to reduced lignin accumulation [53]. Previous studies have demonstrated the positive roles of key enzymes, such as *PAL*, *4CL*, *HCT*, *CYP98A*, and *CCoAOMT* in the [6]-gingerol biosynthetic pathway [54,55,56,57]. Kriegshauser et al. [54] emphasized the crucial and conserved role of *HCT* genes in the phenylpropanoid pathway. Tang et al., [55] demonstrated that the transcript levels of *CCoAOMT* were strongly correlated with [6]-gingerol accumulation in ginger and further validated its regulatory role through virus-induced gene silencing (VIGS).

The observed co-expression between genes such as *HCT* and [6]-gingerol accumulation in G9 suggests a potential metabolic association. However, the specific role of *HCT* in [6]-gingerol biosynthesis remains to be established, as direct evidence from in vitro enzyme assays, metabolite intermediate tracking or isotope labeling, and *in planta* functional validation, such as gene knockdown or overexpression, should be part of future studies. These experiments will be essential to validate the proposed link. Once the underlying mechanism is clarified, strategies such as regulating the expression of key enzyme genes through genetic engineering or optimizing cultivation conditions may be explored to promote [6]-gingerol accumulation in *M. doumeri*. For instance, overexpressing the heterologous α-Zingiberene synthase gene, the sesquiterpene content in wild tomatoes can be increased from less than 0.01 to 0.8–1.5 μg/g FW [58], by controlling water stress, selecting the optimal light ratio, balancing nutrients and other agricultural technologies, the resveratrol content in berries can be increased from 0.03 to 15 μg/g [59]. CRISPR/Cas9 editing of the banana lycopene epsilon-cyclase gene increased the β-carotene content by 6 times (to 24 μg/g FW) [60].

### 3.4. Processing Implications for Functional Metabolites

Given that *M. doumeri* fruit is often dried before consumption or further processing, we conducted a preliminary assessment of how freeze-drying affects the retention of the functional metabolites identified above and their potential applications. Given the widespread use of drying in food preservation and processing, and the heat and oxidation sensitivity of many flavonoids, vacuum freeze-drying minimizes oxidative and thermal degradation while preserving fruit color and shape, thereby improving product quality and marketability [61]. Accordingly, we compared total flavonoid content and antioxidant activity between fresh and freeze-dried G9 samples. The freeze-dried samples showed a total flavonoid content of 72.61 mg/g and achieved > 90% antioxidant activity at 0.15 mg/mL, with no significant difference from VC. Both metrics were significantly higher than those of fresh fruit. This pattern is consistent with processing studies in other horticultural products, including hawthorn, Citrus limon, and Taihang chrysanthemum [62,63,64]. The mechanism lies in the fact that freezing and vacuum conditions effectively prevent the oxidation and thermal degradation of phenolic compounds [62,64]. In addition, freeze-drying can modify cell wall structure and facilitate the release of bound phenolics, thereby increasing the extractability of total flavonoids [61].

These findings suggest that [6]-gingerol content may serve as a reference indicator for germplasm selection and for optimizing processing. For future applications, selecting germplasms with high HCT expression and employing gentle processing methods, such as freeze-drying, may help retain the target compound. However, the bioavailability, long-term stability, and toxicity of [6]-gingerol in processed *M. doumeri* products were not evaluated in this study and warrant further investigation.

## 4. Materials and Methods

### 4.1. Plant Materials

Fruit samples of *M. doumeri* were collected at the core production base of the large-hawthorn (*M. doumeri*) geographical indication agricultural product protection project in Yishu, Jingxi County, Baise, Guangxi Zhuang Autonomous Region, China (Lat. 23°34′ N, Long. 106°28′ E, Alt. 798.8 m). This base serves as a germplasm resource nursery managed by Guangxi Jingxi Liangpeng Food Co., Ltd. in Jingxi, China, with well-documented varietal origins. All plants used were grafted seedlings commonly grown in commercial cultivation. For each germplasm, over 20 fruits of uniform size and free from mechanical or insect disease were randomly collected from the upper-middle canopy of the selected trees, based on morphological features and ~90% maturity. To ensure sample traceability, each specimen was labeled on-site, and corresponding records and photographic documentation were retained. The samples were rinsed with ultrapure water and 70% ethanol (Macklin, Shanghai, China) to remove surface contaminants. The samples were deseeded and cut into pieces, combined and homogenized, and then divided into fresh and freeze-dried groups. After flash-freezing in liquid nitrogen (Macklin, Shanghai, China), the fresh group was stored at −80 °C until further processing. The freeze-dried group was pre-chilled at −80 °C for 1 h, then freeze-dried at −60 °C under a vacuum of <10 Pa for 48 h using a freeze dryer (ZLGJ-10, Hebei Yingsheng Instrument Equipment Co., Ltd., Hengshui, China) until constant weight. Dried samples were sealed and stored at −80 °C for further use.

### 4.2. Determination of Total Flavonoids

Three biological replicates were prepared for each *M. doumeri* germplasm, with samples processed as described in Section 4.1. Fresh and freeze-dried samples were subjected to ultrasonic extraction with 70% ethanol at a material-to-liquid ratio of 1:18 for 30 min at 300 W. This process was repeated twice. The resulting supernatant was collected. Total flavonoid content was measured using the aluminum nitrate colorimetric method, according to Matić et al. [65]. Absorbance was measured at 510 nm using a Tecan Infinite 2000pro multimode microplate reader (Männedorf, Switzerland), and a standard calibration curve was developed. In preliminary work, the total flavonoid content was compared among various *M. doumeri* germplasms (Figure S1). Two germplasms, G8 and G9, demonstrated significant differences in their total flavonoid content and were selected for further analysis and comparison.

### 4.3. Analysis of Flavonoids Metabolites by Non-Targeted Metabolomics and Quantification of [6]-Gingerol

Three biological replicates were prepared per germplasm as described in Section 4.1. Samples preparation, extract analysis, metabolite identification, and relative quantification were conducted by VeryGenome Technology Co., Ltd., Guangzhou, China in accordance with their standardized protocols and established methodologies. Methanol and acetonitrile were purchased from Honeywell (Shanghai, China), and ammonium acetate was purchased from Sigma-Aldrich (St. Louis, MO, USA). Briefly, fruit powder was extracted with methanol: acetonitrile: water (2:2:1, *v*/*v*/*v*). After sonication in an ice-water bath, the extract was flash-frozen in liquid nitrogen and centrifuged, and the supernatant was collected, dried under nitrogen, and reconstituted in acetonitrile: water (1:1, *v*/*v*). The reconstituted solution was sonicated again in an ice-water bath, centrifuged, and the final supernatant was subjected to mass spectrometry analysis.

An ultra-high-performance liquid chromatography-mass spectrometry (UPLC-MS) system, equipped with a Waters UPLC BEH Amide column (Milford, MA, USA) (2.1 × 100 mm, 1.7 μm), was used for liquid chromatography separations and mass spectrometry detection. An injection volume of 5 μL was utilized under specific conditions, with the column temperature (55 °C). The mobile phase gradient included water with 25 mM ammonium acetate and 25 mM ammonium hydroxide (Phase A) along with 100% acetonitrile (Phase B). The gradient was programmed as follows: from 0 to 1 min, 85% B; from 1 to 12 min, 65% B; from 12 to 12.1 min, 40% B; from 12.1 to 15 min, 40% B; from 15 to 15.1 min, 85% B; and finally, from 15.1 to 20 min, 85% B. A flow rate of 0.3 mL/min was maintained. Mass spectrometry data were acquired using electrospray ionization (ESI) with an ion source voltage of 4500 V or 5500 V, curtain gas at 20 psi, and nebulizing and auxiliary gases at 60 psi each. Raw UPLC-MS data were converted to mzXML format using ProteoWizard, followed by peak alignment, retention time correction, and peak area integration using SCIEX OS. Metabolite identification was performed by matching both primary and secondary spectra (within 25 ppm) against the proprietary VeryGenome database and public metabolomic databases. For quality control and data processing, a pooled QC sample was injected after every eight experimental samples throughout the analytical batch. Instrument stability was assessed by confirming the close overlap of total ion chromatograms (TICs) across all QC injections and ensuring that the relative standard deviation (RSD) of internal standards in the QC samples remained below 15%. Metabolite features with an RSD greater than 30% in the QC samples were removed before downstream analysis. The filtered peak area data were normalized using QC-based robust LOESS signal correction to minimize signal drift. After normalization, features with more than 50% missing values across all samples were excluded. Remaining missing values were imputed using half of the minimum detected value for the corresponding feature. Metabolite quantification was performed in multiple reaction monitoring (MRM) mode, and the normalized peak areas were used for relative quantification.

Flavonoids were compared with the metabolic features of G8 and G9 using orthogonal partial least squares discriminant analysis (OPLS-DA) and hierarchical clustering analysis (HCA). Metabolites match all three criteria (variable importance in projection (VIP) score ≥ 1, |log_2_FC| ≥ 1, and *p* < 0.05) were identified as significantly differentially accumulated metabolites. False discovery rate (FDR) correction was applied, and the three criteria described above were used for final selection.

Absolute quantification of [6]-gingerol in samples G8 and G9 was performed using external standard calibration by VeryGenome Technology Co., Ltd. The [6]-gingerol standard (MedChemExpress, Shanghai, China) was prepared into a gradient working solution with methanol. The sample pretreatment and detection methods were referred to 4.3. The peak areas of the samples were quantified to obtain the total peak area. The sample peak area was substituted into the linear regression equation to calculate the concentration, and the result was then converted to dry weight content (μg/g).

### 4.4. RNA-Sequencing Analysis

Three biological replicates per germplasm (Section 4.1) were prepared for RNA sequencing (RNA-Seq), which was conducted at VeryGenome Technology Co., Ltd. Total RNA was extracted from the tissues of *M. doumeri* using TRIzol reagent (Invitrogen, Thermo Fisher Scientific, Waltham, MA, USA), and the quality of the RNA was initially assessed with a NanoDrop 2000 spectrophotometer (Thermo Fisher Scientific, Waltham, MA, USA), agarose gel electrophoresis and Agilent 2100 Bioanalyzer (Agilent Technologies, Inc., San Diego, CA, USA). Messenger RNA (mRNA) was enriched using magnetic beads attached to oligo(dT) (Invitrogen, Thermo Fisher Scientific, Waltham, MA, USA), followed by fragmentation with a fragmentation buffer. cDNA libraries were constructed using the TruSeq™ RNA Sample Preparation Kit (Illumina, San Diego, CA, USA). The products that ligated to adapters were purified and size-selected with Agencourt AMPure XP beads, followed by PCR amplification. The libraries were purified and quantified using the Quantus™ Fluorometer and QuantiFluor^®^ dsDNA System for quality control (Promega, Madison, WI, USA). Sequencing was performed using Illumina HiSeq Xten, NovaSeq 6000, or MGI T7 platforms (MGI Tech Co., Ltd., Shenzhen, China).

Quality control of the raw sequencing data was conducted using fastp (v0.23.4). The clean reads were then aligned to the reference genome of *Malus domestica* (PRJNA1194184) using HiSat2 and TopHat2. Alignment quality metrics, including sequencing saturation, gene coverage, read distribution across genomic regions, and the chromosomal distribution of mapped reads, were evaluated to assess data quality [66,67]. Gene and transcript expression levels were quantified using StringTie (v2.2.3) [68]. Differential gene expression analysis between samples was performed using edgeR (v4.2.0) and DEGseq (v1.56.0) [69]. Genes were defined as differentially expressed genes (DEGs) if they had |log_2_FC| ≥ 1, and padj < 0.05.

Gene Ontology (GO) enrichment analysis of DEGs was performed using GOATOOLS (v1.3.0). with statistical significance evaluated by Fisher’s exact test. To control for multiple testing, we applied Bonferroni, Holm, Sidak, and false discovery rate (FDR) adjustments to the *p*-values. Gene Ontology (GO) terms with adjusted *p*-values (p-fdr) of 0.05 or lower were deemed significantly enriched [70]. KEGG pathway enrichment analysis was conducted using KOBAS (v3.0.3), with statistical significance evaluated through Fisher’s exact test. To control the false positive rate, multiple testing correction was performed using the Benjamini-Hochberg (FDR) method. KEGG pathways with an adjusted *p*-value (FDR) ≤ 0.05 were significantly enriched among the differentially expressed genes [71].

Transcription factors that were differentially expressed identified using the Plant Transcription Factor Database (PlantTFDB) and the Animal Transcription Factor Database (AnimalTFDB). The selection criteria for these differentially expressed transcription factors were based on |log_2_FC| ≥ 1, and padj < 0.05.

### 4.5. Real-Time Quantitative PCR Validation and Quantification of [6]-Gingerol

RNA extraction and cDNA synthesis were carried out as described previously. Three biological replicates were used for each group. In light of transcriptomics results, nine genes involved in flavonoid biosynthesis were selected for validation using quantitative real-time PCR (qRT-PCR). Gene-specific primers were designed using Primer Premier 5 software, with β-actin used as the reference gene (Table S1). The qRT-PCR reactions were prepared using the Ultra SYBR Mix system (Vazyme, Nanjing, China) in a total volume of 20 μL. qRT-PCR was performed using a QuantReady Real-Time PCR System (K9600, Hangzhou Suizhen Biotechnology Co., Ltd., Hangzhou, China). The amplification program included an initial denaturation step at 95 °C for 30 s, followed by 40 cycles consisting of denaturation at 95 °C for 10 s and annealing/extension at 60 °C for 30 s. Relative gene expression levels were calculated using the 2^−ΔΔCT^ method.

### 4.6. Antioxidant Activity of M. doumeri Extracts

The antioxidant activity was assessed using 1,1-Diphenyl-2-picrylhydrazyl (DPPH·) and ·OH assays following H. Liu et al. [64] with minor modifications. All reagents used in this section were purchased from Macklin (Shanghai, China). The *M. doumeri* extract solution from Section 4.2 was diluted to a series of concentrations in 70% ethanol, with four biological replicates at each concentration. Sample solutions (90 μL) at various concentrations were mixed with 90 μL 0.1 mmol/L DPPH solution (A1). The control group (A2), used anhydrous ethanol instead of DPPH, and the blank group (A0) contained ethanol and DPPH without a sample. VC served as a positive control. After 20 min of incubation at room temperature, absorbance was measured at 517 nm, and the DPPH· radical scavenging activity (%) was calculated as:

DPPH· radical scavenging activity (%) = [(1 − (A1 − A2))/A0] × 100

Hydroxyl radical (·OH) scavenging activity was measured using the Fenton reaction method described by Bibi et al. [72] with minor modifications. The reaction mixture contained 0.2 mL of 1.5 mmol/L 1,10-phenanthroline hydrate, 0.6 mL of phosphate-buffered saline (pH 7.4), 0.2 mL of 1.5 mmol/L FeSO_4_, 0.2 mL of 0.03% H_2_O_2_, and 0.2 mL of the sample solution. The total volume was adjusted to 1.4 mL with distilled water (A1). Control mixtures were prepared in parallel, including A2 (without sample) and A0 (without sample and H_2_O_2_). VC was used as positive control. After 1 h incubation at 37 °C, absorbance was measured at 510 nm and calculations were performed using following equation.

·OH radical scavenging activity (%) = [((A1 − A2))/((A0 − A2))] × 100

### 4.7. Statistical Analysis

Statistical analyses were performed using GraphPad Prism (v10.1.2) and R (v4.4.3). Differences between two groups were assessed using a two-tailed Student’s *t*-test, whereas multiple groups were compared using one-way analysis of variance (ANOVA) followed by Tukey’s multiple range test. *p* < 0.05 was considered statistically significant. Multivariate statistical analyses were performed using the Metware Cloud platform (https://cloud.metware.cn).

## 5. Conclusions

This study compared the metabolomic and transcriptomic profiles of two large-fruited hawthorn (*M. doumeri*) germplasms (G8 and G9) to characterize functional metabolites and elucidate the molecular basis of their biosynthesis. The major and differentially accumulated metabolites were mainly flavonoids and isoflavonoids. Germplasm with high-flavonoid content (G9) showed distinct metabolic and transcriptional features. Integrated analysis indicated that the differentially expressed genes were enriched in phenylpropanoid biosynthesis and flavone and flavonol biosynthesis pathways. Several structural genes and transcription factors potentially associated with the biosynthesis of [6]-gingerol and related metabolites were also identified. qRT-PCR and absolute quantification supported the transcriptomic and metabolomic trends, while freeze-drying preserved high metabolite contents and antioxidant activity. These findings suggest that flavonoid accumulation in *M. doumeri* is closely linked to coordinated regulation of phenylpropanoid-related pathways and provide candidate molecular targets for germplasm evaluation, breeding, and specialty product development. However, the proposed regulatory mechanisms require functional validation, and their generalizability should be tested across a broader germplasm pool. Future studies should verify candidate gene functions, clarify pathway regulation, and integrate cultivation and processing optimization. Overall, this study provides a useful multi-omics foundation for the nutritional evaluation and value-added utilization of large-fruited hawthorn.

## Data Availability

The original contributions presented in this study are included in the Article/Supplementary Materials. Further inquiries can be directed to the corresponding author.

## Supplementary Materials

The following supporting information can be downloaded at: https://www.mdpi.com/article/10.3390/molecules31111857/s1, Table S1: Primer sequences of genes used for quantitative qRT-PCR verification; Table S2: Summary statistics of RNA-Seq results in *M. doumeri*; Table S3: List of differentially expressed structural genes of flavonoids in related pathways in G8 and G9; Table S4: The KEGG pathway enriched with differential flavonoid metabolites in the transcriptomic-metabolomic co-analysis; Table S5: The screened information of key transcription factors; Table S6: The qRT-PCR and RNA-Seq data of G8 and G9 related genes were compared based on the Pearson correlation coefficient; Table S7: Absolute quantitative chromatograms of G8 and G9; Table S8: Identification and validation data for [6]-gingerol; Figure S1: Mature fruits of *M. doumeri* various germplasms in preliminary work (A), and comparison of total flavonoid content among these germplasms (B). Different lowercase letters indicate significant differences in flavonoid content in Tukey’s multiple comparison test (*p* < 0.05); Figure S2: Extracted ion chromatogram (EIC) of authentic [6]-gingerol standard (A); mirror plot of MS/MS spectra of [6]-gingerol (B).

## Abbreviations

The following abbreviations are used in this manuscript:

*Malus doumeri* (Bois) Chev.	*M. doumeri*
Phenylalanine ammonia-lyase	*PAL*
4-Coumarate-CoA ligase	*4CL*
Cinnamate 4-hydroxylase	*CYP73A*
Hydroxycinnamoyl-CoA shikimate/Quinate hydroxycinnamoyl transferase	*HCT*
Caffeoyl-CoA O-methyltransferase	*CCoAOMT*
Cytochrome P450 98A	*CYP98A*
