# Supplementary material for: Comparative Metabolomic and Transcriptomic Analyses Identify Candidate Genes Associated with Flavonoid Accumulation and Phenylpropanoid Metabolism in Large-Fruited Hawthorn (Malus doumeri (Bois) Chev.)
Source: Molecules. 2026 May 28;31(11):1857. doi: 10.3390/molecules31111857 (PMC13257776; doi:10.3390/molecules31111857)
Supplement: Supplementary file 1 [file molecules-31-01857-s001.zip › molecules-4284446-supplementary.pdf]

## Supplementary Materials

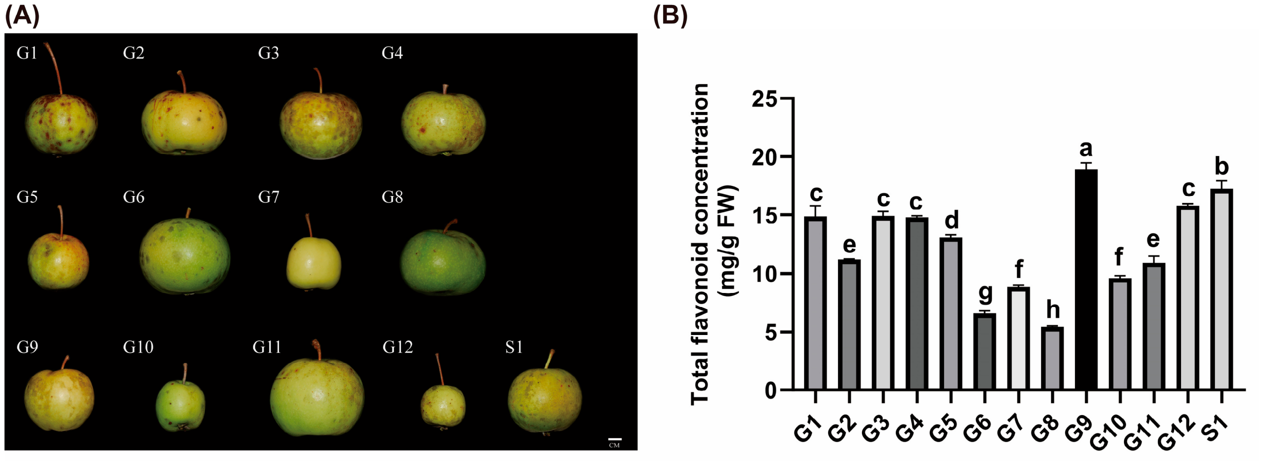


**Figure S1.** Mature fruits of *M. doumeri* various germplasms in preliminary work (A), and comparison of total flavonoid content among these germplasms (B). Different lowercase letters indicate significant differences in flavonoid content in Tukey's multiple comparison test ($P < 0.05$).

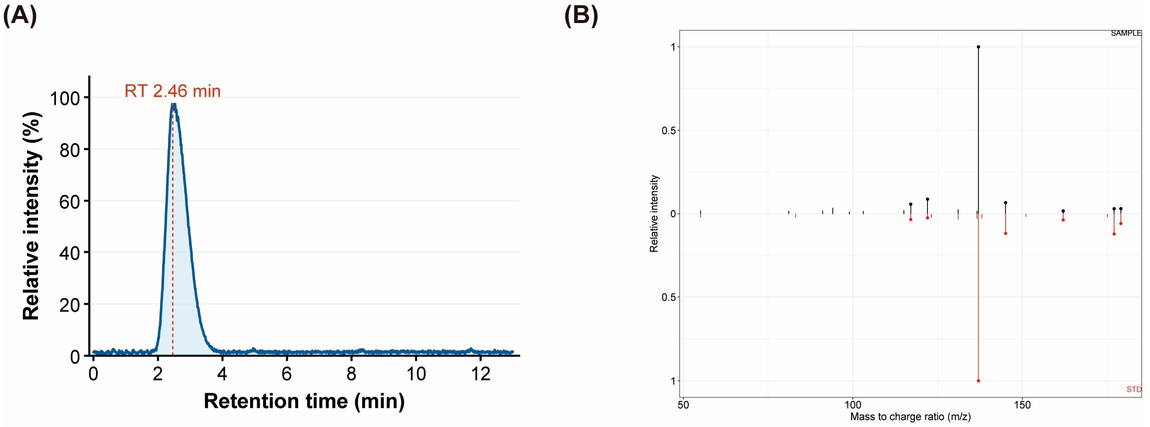


**Figure S2.** Extracted ion chromatogram (EIC) of authentic [6]-gingerol standard (A); mirror plot of MS/MS spectra of [6]-gingerol (B).

**Table S1.** Primer sequences of genes used for quantitative qRT-PCR verification.

| Gene Name | Gene ID | Forward Primer (5'→3') | Reverse Primer (5'→3') |
|---|---|---|---|
| *β-actin* | LOC103453508 | TGGTTGGTATGGGTCAGAAGG | CTGTGAGCAGAACTGGGTGTT |
| *HCT* | LOC103405591 | GCCATCTTCTGCTAATCCAGGCCAA | TGGCAGGAGGAGGTGAGTT |
| *HCT* | LOC114821133 | ATCCACTTGGCGGTCCTCTA | TCTCTTCGATCTTCGTCATGGT |
| *HCT* | LOC114821135 | TTAGACATTTGTGCCTCTCCCT | CGTTATTTTTGAATTCCCCTGC |
| *HCT* | LOC103403337 | TGCCACTGCAACTCCAACG | CTTCCCCCAACCAAAATCA |

| | | | |
|---|---|---|---|
| *HCT* | LOC103409539 | CTTCTTTGAATGTGGCGGG | TGGCAGGAGGAGGTGAGTT |
| *HCT* | LOC103454980 | CCTAAAAATTGCCGCCCTC | AACTCTATCTCACCATCCTCCTCT |
| *4CL* | LOC103426517 | GCTCTATTAGGGTGGTGTTGTCT | GACTTGGTTGGCATCGGTT |
| *WRKY* | LOC103427630 | ATTTCTGTGCCTACGCCTCC | CGCCACTCTAATCGTCCTCTT |
| *MYB* | LOC103434665 | AAGGTTGACACCAGGAAGCG | CCAAGCCAGCAAAGAAGGAC |
| *bHLH* | LOC103422512 | TGGGTTCCTGTGTCTATCTTGC | CAGCTTTCTGGTTTCTGCCTT |

**Table S2.** Summary statistics of RNA-Seq results in *M. doumeri*.

| Samples | Total Reads | Clean Reads | Percentage of Clean Reads (%) | Total bases | Clean bases | GC Content (%) | %>Q20 | %>Q30 | Mapped Reads (%) | Unique alignments (%) | Secondary alignments (%) |
|---|---|---|---|---|---|---|---|---|---|---|---|
| G8-1 | 39,900,830 | 39,771,488 | 99.68 | 5,985,124,500 | 5,948,910,422 | 47.47 | 99.10 | 96.59 | 32,564,436(81.88) | 31,098,325(78.19) | 1,466,111(3.69) |
| G8-2 | 45,007,942 | 45,007,942 | 100.00 | 6,751,191,300 | 6,701,115,996 | 47.36 | 99.40 | 97.49 | 36,386,750(80.85) | 35,002,586(77.77) | 1,384,164(3.08) |
| G8-3 | 52,149,940 | 52,149,940 | 100.00 | 7,822,491,000 | 7,800,636,246 | 47.25 | 99.46 | 97.67 | 42,325,122(81.16) | 40,431,097(77.53) | 1,894,025(3.63) |
| G9-1 | 37,883,746 | 37,883,746 | 100.00 | 5,682,561,900 | 5,666,990,638 | 46.86 | 99.42 | 97.50 | 30,623,818(80.84) | 29,583,909(78.09) | 1,039,909(2.75) |
| G9-2 | 59,064,114 | 58,434,820 | 98.93 | 8,859,617,100 | 8,742,992,990 | 47.03 | 98.39 | 95.17 | 47,768,965(81.75) | 46,072,192(78.84) | 1,696,773(2.9) |
| G9-3 | 47,774,756 | 47,774,756 | 100.00 | 7,166,213,400 | 7,136,159,038 | 46.17 | 99.66 | 98.51 | 39,381,397(82.43) | 37,986,751(79.51) | 1,394,646(2.92) |

**Table S3.** List of differentially expressed structural genes of key metabolites in related pathways in G8 and G9.

| Gene Name | Gene ID | G8 Mean FPKM | G9 Mean FPKM | Log2FC | padj | G8 Vs. G9 up / down | Enriched KEGG |
|---|---|---|---|---|---|---|---|
| *CAD* | LOC103405068 | 68.00 | 27.67 | 1.58E+00 | 1.93E-02 | up | Phenylpropanoid biosynthesis ko00940 |
| *CAD* | LOC103405070 | 175.33 | 86.00 | 1.36E+00 | 9.02E-04 | up | |
| *UGT72E* | LOC103455522 | 141.67 | 17.33 | 3.26E+00 | 5.63E-05 | up | |
| *COMT* | LOC103439958 | 343.00 | 65.67 | 2.64E+00 | 4.95E-05 | up | |
| *COMT* | LOC103439956 | 457.33 | 84.67 | 2.65E+00 | 2.64E-06 | up | |
| *COMT* | LOC103405809 | 181.67 | 87.00 | 1.38E+00 | 2.21E-02 | up | |
| *4CL* | LOC103427406 | 1198.00 | 635.33 | 1.23E+00 | 2.43E-06 | up | |
| *K22395* | LOC114824987 | 65.33 | 25.33 | 1.73E+00 | 4.72E-02 | up | |
| *K22395* | LOC114824988 | 80.00 | 13.67 | 2.83E+00 | 1.46E-03 | up | |
| *CCR* | LOC103429871 | 5051.00 | 2597.33 | 1.18E+00 | 8.84E-05 | up | |
| *CCR* | LOC103413149 | 197318.33 | 83222.67 | 1.56E+00 | 9.78E-15 | up | |
| *E1.11.1.7* | LOC103417852 | 1741.00 | 842.33 | 1.28E+00 | 6.41E-06 | up | |
| *HCT* | LOC103409539 | 218.00 | 1045.67 | -1.94E+00 | 4.30E-02 | down | |

| | | | | | | | |
|---|---|---|---|---|---|---|---|
| *HCT* | LOC103405591 | 170.33 | 485.00 | -1.23E+00 | 2.95E-05 | down | |
| *HCT* | LOC103403337 | 296.67 | 763.00 | -1.02E+00 | 5.45E-05 | down | |
| *HCT* | LOC114821135 | 148.67 | 493.33 | -1.37E+00 | 8.79E-06 | down | |
| *HCT* | LOC114821133 | 51.33 | 173.33 | -1.45E+00 | 1.60E-03 | down | |
| *HCT* | LOC103454980 | 662.67 | 2063.67 | -1.36E+00 | 1.84E-08 | down | |
| *COMT* | LOC103426499 | 4.67 | 46.33 | -3.00E+00 | 9.78E-03 | down | |
| *4CL* | LOC103426517 | 85.67 | 224.33 | -1.08E+00 | 2.73E-02 | down | |
| *CYP84A, F5H* | LOC103436113 | 10.00 | 70.00 | -2.46E+00 | 4.82E-02 | down | |
| *K22395* | LOC114823254 | 14.33 | 234.33 | -3.58E+00 | 4.50E-05 | down | |
| *K22395* | LOC103455003 | 348.33 | 1108.67 | -1.30E+00 | 1.12E-05 | down | |
| *CCR* | LOC103412876 | 19.33 | 76.67 | -1.76E+00 | 1.63E-02 | down | |
| *E1.11.1.7* | LOC103427044 | 122.00 | 339.33 | -1.14E+00 | 1.74E-02 | down | |
| *E1.11.1.7* | LOC103418409 | 12.33 | 218.33 | -3.99E+00 | 9.43E-03 | down | |
| *E1.11.1.7* | LOC103407572 | 89.67 | 423.33 | -1.92E+00 | 1.08E-05 | down | |
| *E1.11.1.7* | LOC103417791 | 4.00 | 62.00 | -3.51E+00 | 1.66E-03 | down | |
| *FLS* | FLS | 2103.67 | 817.67 | 6.80E-16 | 9.93E-14 | up | |
| *FLS* | LOC103441195 | 5120.67 | 3120.33 | 1.47E-07 | 4.56E-06 | up | |
| *FLS* | LOC103452933 | 8609.00 | 4705.33 | 1.02E-22 | 3.49E-20 | up | |
| *C12RT1* | LOC103408464 | 1458.33 | 26.33 | 1.93E-14 | 2.28E-12 | up | |
| *C12RT1* | LOC103407571 | 215.33 | 133.00 | 1.85E-03 | 1.41E-02 | up | |
| *C12RT1* | LOC103407359 | 5013.67 | 89.67 | 4.38E-12 | 3.49E-10 | up | |
| *C12RT1* | LOC103438843 | 1640.00 | 921.67 | 9.04E-06 | 1.66E-04 | up | |
| *PGT1* | LOC103425310 | 16186.00 | 9778.33 | 1.91E-16 | 3.08E-14 | up | Flavonoid biosynthesis ko00941 |
| *PGT1* | LOC103402192 | 232706.67 | 143693.67 | 3.46E-13 | 3.37E-11 | up | |
| *HCT* | LOC103409539 | 218.00 | 1045.67 | 7.54E-03 | 4.30E-02 | down | |
| *HCT* | LOC103405591 | 170.33 | 485.00 | 1.25E-06 | 2.95E-05 | down | |
| *HCT* | LOC103403337 | 296.67 | 763.00 | 2.52E-06 | 5.45E-05 | down | |
| *HCT* | LOC114821135 | 148.67 | 493.33 | 3.13E-07 | 8.79E-06 | down | |
| *HCT* | LOC114821133 | 51.33 | 173.33 | 1.30E-04 | 1.60E-03 | down | |
| *HCT* | LOC103454980 | 662.67 | 2063.67 | 3.26E-10 | 1.84E-08 | down | |
| *LAR* | LAR1 | 723.00 | 2919.67 | 2.76E-10 | 1.58E-08 | down | |
| *CYP81E* | LOC103432708 | 1748.67 | 896.67 | 9.33E-09 | 3.84E-07 | up | Isoflavonoid biosynthesis |

| | | | | | | | |
|---|---|---|---|---|---|---|---|
| *PTS* | LOC103447015 | 62.00 | 204.33 | 9.08E-04 | 8.01E-03 | down | ko00943 |
| *C12RT1* | LOC103408464 | 1458.33 | 26.33 | 1.93E-14 | 2.28E-12 | up | Flavone and flavonol biosynthesis ko00944 |
| *C12RT1* | LOC103407571 | 215.33 | 133.00 | 1.85E-03 | 1.41E-02 | up | |
| *C12RT1* | LOC103407359 | 5013.67 | 89.67 | 4.38E-12 | 3.49E-10 | up | |
| *C12RT1* | LOC103438843 | 1640.00 | 921.67 | 9.04E-06 | 1.66E-04 | up | |

Note: The “up” and “down” designations in the “G8 Vs. G9” columns refer to the direction of change in the G8 vs. G9 comparison. Therefore, “down” in these columns indicates higher expression or abundance in G9.

**Table S4.** The KEGG pathway enriched with differential key metabolites in the transcriptomic-metabolomic co-analysis.

| Enriched KEGG | Metabolite Name | Gene Name | GeneID | genes G8 Vs. G9 | Metabolites G8 Vs. G9 | Correlation | *P*-value |
|---|---|---|---|---|---|---|---|
| Degradation of flavonoids ko00946 | Genistin | *bglB/ bglX* | LOC103427795 | down | up | -0.7232 | 0.1043 |
| | | | LOC103418926 | up | | 0.4154 | 0.4127 |
| | Apigenin | | LOC103427795 | down | down | 0.7741 | 0.0708 |
| | | | LOC103418926 | up | | -0.2938 | 0.5720 |
| Stilbenoid, diarylheptanoid and gingerol biosynthesis ko00945 | [6]-Gingerol | *HCT* | LOC103409539 | down | down | 0.8102 | 0.0506 |
| | [6]-Gingerol | *HCT* | LOC103405591 | down | down | 0.8693 | 0.0245 |
| | [6]-Gingerol | *HCT* | LOC103403337 | down | down | 0.9043 | 0.0132 |
| | [6]-Gingerol | *HCT* | LOC114821135 | down | down | 0.9707 | 0.0013 |
| | [6]-Gingerol | *HCT* | LOC114821133 | down | down | 0.9216 | 0.0089 |
| | [6]-Gingerol | *HCT* | LOC103454980 | down | down | 0.8635 | 0.0267 |
| Flavone and flavonol biosynthesis ko00944 | Apigenin | *C12RT1* | LOC103408464 | up | down | -0.7125 | 0.1121 |
| | Apigenin | *C12RT1* | LOC103407571 | up | down | -0.6873 | 0.1314 |
| | Apigenin | *C12RT1* | LOC103407359 | up | down | -0.6821 | 0.1355 |
| | Apigenin | *C12RT1* | LOC103438843 | up | down | -0.6759 | 0.1406 |
| Isoflavonoid biosynthesis ko00943 | Glycitin | *PTS/ CYP 81E* | LOC103447015 | down | up | -0.9357 | 0.0061 |
| | | | LOC103432708 | up | | 0.6049 | 0.2033 |
| | Genistin | | LOC103447015 | down | up | -0.7611 | 0.0788 |
| | | | LOC103432708 | up | | 0.8085 | 0.0515 |
| | Glycitein | | LOC103447015 | down | up | -0.9447 | 0.0045 |
| | | | LOC103432708 | up | | 0.6209 | 0.1883 |
| | Apigenin | | LOC103447015 | down | down | 0.7641 | 0.0769 |
| | | | LOC103432708 | up | | -0.7149 | 0.1103 |
| Flavonoid biosynthesis ko00941 | Apigenin | *HCT* | LOC103409539 | down | down | 0.6501 | 0.1622 |
| | Apigenin | *HCT* | LOC103405591 | down | down | 0.8035 | 0.0541 |
| | Apigenin | *HCT* | LOC103403337 | down | down | 0.9392 | 0.0054 |
| | Apigenin | *HCT* | LOC114821135 | down | down | 0.9524 | 0.0033 |
| | Apigenin | *HCT* | LOC114821133 | down | down | 0.8581 | 0.0288 |
| | Apigenin | *HCT* | LOC103454980 | down | down | 0.6836 | 0.1343 |
| | Apigenin | *LAR* | LAR1 | down | down | 0.9109 | 0.0116 |
| | Apigenin | *FLS* | FLS | up | down | -0.6768 | 0.1398 |
| | Apigenin | *FLS* | LOC103441195 | up | down | -0.6862 | 0.1323 |
| | Apigenin | *FLS* | LOC103452933 | up | down | -0.8019 | 0.0549 |
| | Apigenin | *C12RT1* | LOC103408464 | up | down | -0.7125 | 0.1121 |
| | Apigenin | *C12RT1* | LOC103407571 | up | down | -0.6873 | 0.1314 |

| | | | | | | |
|---|---|---|---|---|---|---|
| Apigenin | *C12RT1* | LOC103407359 | up | down | -0.6821 | 0.1355 |
| Apigenin | *C12RT1* | LOC103438843 | up | down | -0.6759 | 0.1406 |
| Apigenin | *PGT1* | LOC103425310 | up | down | -0.7343 | 0.0965 |
| Apigenin | *PGT1* | LOC103402192 | up | down | -0.6925 | 0.1273 |

Note: The "up" and "down" designations in the "G8 Vs. G9" columns refer to the direction of change in the G8 vs. G9 comparison. Therefore, "down" in these columns indicates higher expression or abundance in G9.

**Table S5.** The screened information of key transcription factors

| TF Family | Gene ID | Log2FC | padj | G8 Vs. G9 up / down |
|---|---|---|---|---|
| *WRKY* | LOC103427630 | -1.94E+00 | 9.31E-10 | down |
| *MYB* | LOC103434665 | -6.13E+00 | 8.12E-03 | down |
| *bHLH* | LOC103422512 | -5.42E+00 | 4.97E-02 | down |

Note: The "up" and "down" designations in the "G8 Vs. G9" columns refer to the direction of change in the G8 vs. G9 comparison. Therefore, "down" in these columns indicates higher expression or abundance in G9.

**Table S6.** The qRT-PCR and RNA-Seq data of G8 and G9 related genes were compared based on the Pearson correlation coefficient.

| Gene Name | Gene ID | Correlation | *P*-value |
|---|---|---|---|
| *HCT* | LOC103405591 | 0.5650 | 0.2426 |
| *HCT* | LOC114821133 | 0.9482 | 0.0040 |
| *HCT* | LOC114821135 | 0.6721 | 0.1437 |
| *HCT* | LOC103403337 | 0.9389 | 0.0055 |
| *HCT* | LOC103409539 | 0.3689 | 0.4718 |
| *HCT* | LOC103454980 | 0.8951 | 0.0159 |
| *4CL* | LOC103426517 | 0.3013 | 0.5618 |
| *WRKY* | LOC103427630 | 0.8380 | 0.0372 |
| *MYB* | LOC103434665 | 0.7717 | 0.0722 |
| *bHLH* | LOC103422512 | 0.8762 | 0.0220 |

**Table S7.** Absolute quantitative chromatograms of G8 and G9.

| Sample | Area | Content (μg/g) |
|---|---|---|
| Freeze-dried-G8 | 1.136e+05 | 0.0227 |
| Freeze-dried-G9 | 1.313e+06 | 0.2625 |
| Fresh-G8 | 1.171e+05 | 0.0234 |
| Fresh-G9 | 2.001e+05 | 0.0400 |

Note: Quantification was performed using external standard calibration. The calibration equation was y = 2E-07x ($R^2$ = 0.994), where x is peak area and y is concentration (μg/g).

## **Table S8.** Identification and validation data for [6]-gingerol.

**Analyte: [6]-gingerol (295.2 / 137.1)**

G8-1

RT: 2.47 min

Area: 2.284e4

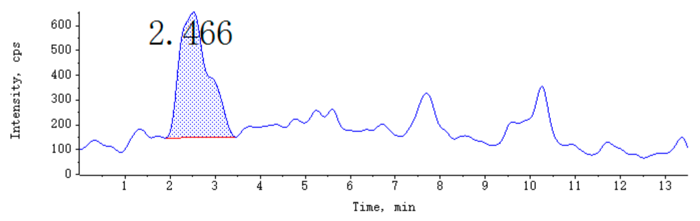


G8-2

RT: 2.50 min

Area: 2.197e4

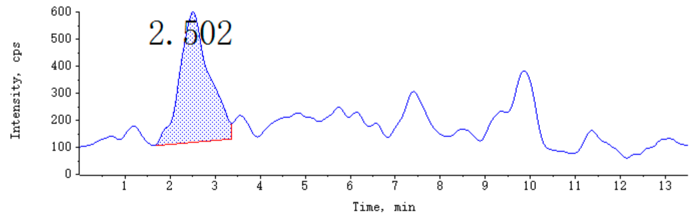


G8-3

RT: 2.40 min

Area: 2.639e4

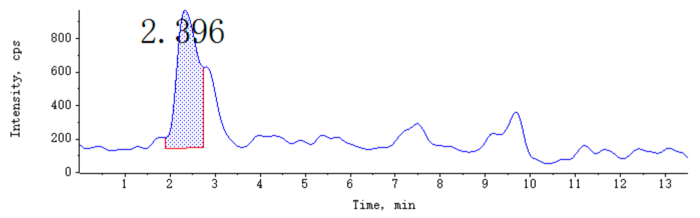


G9-1

RT: 2.34 min

Area: 4.554e4

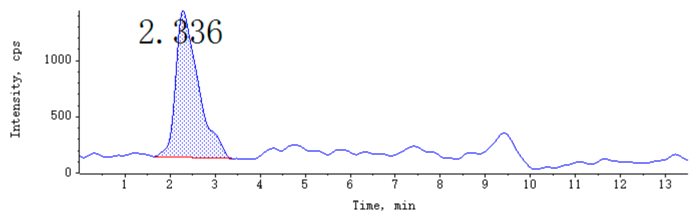


G9-2

RT: 2.33 min

Area: 6.283e4

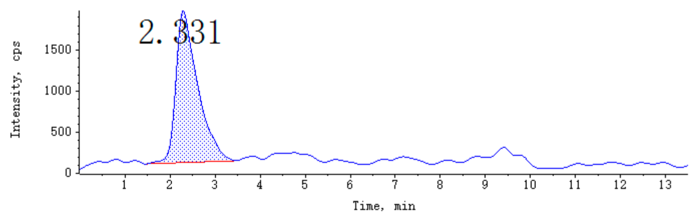

| | |
|---|---|
| G9-3<br>RT: 2.46 min<br>Area: 3.914e4 | 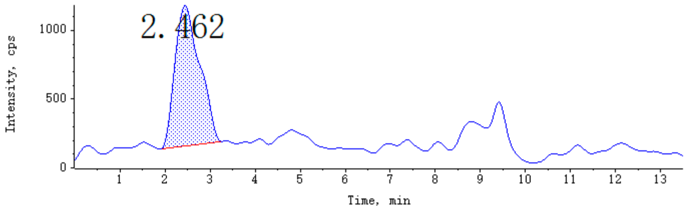 |
